# “Finding an Emotional Face” Revisited: Differences in Own-Age Bias and the Happiness Superiority Effect in Children and Young Adults

**DOI:** 10.3389/fpsyg.2021.580565

**Published:** 2021-03-29

**Authors:** Andras N. Zsido, Nikolett Arato, Virag Ihasz, Julia Basler, Timea Matuz-Budai, Orsolya Inhof, Annekathrin Schacht, Beatrix Labadi, Carlos M. Coelho

**Affiliations:** ^1^Institute of Psychology, University of Pecs, Pecs, Hungary; ^2^Department of Affective Neuroscience and Psychophysiology, Georg-August-Universität Göttingen, Göttingen, Germany; ^3^School of Psychology, ISMAI University Institute of Maia, Maia, Portugal; ^4^Department of Psychology, Faculty of Psychology, Chulalongkorn University, Bangkok, Thailand

**Keywords:** visual search advantage, anger superiority, happiness superiority, own-age bias, children and adults, emotional expressions

## Abstract

People seem to differ in their visual search performance involving emotionally expressive faces when these expressions are seen on faces of others close to their age (peers) compared to faces of non-peers, known as the own-age bias (OAB). This study sought to compare search advantages in angry and happy faces detected on faces of adults and children on a pool of children (*N* = 77, mean age = 5.57) and adults (*N* = 68, mean age = 21.48). The goals of this study were to (1) examine the developmental trajectory of expression recognition and (2) examine the development of an OAB. Participants were asked to find a target face displaying an emotional expression among eight neutral faces. Results showed that children and adults found happy faces significantly faster than angry and fearful faces regardless of it being present on the faces of peers or non-peers. Adults responded faster to the faces of peers regardless of the expression. Furthermore, while children detected angry faces significantly faster compared to fearful ones, we found no such difference in adults. In contrast, adults detected all expressions significantly faster when they appeared on the faces of other adults compared to the faces of children. In sum, we found evidence for development in detecting facial expressions and also an age-dependent increase in OAB. We suggest that the happy face could have an advantage in visual processing due to its importance in social situations and its overall higher frequency compared to other emotional expressions. Although we only found some evidence on the OAB, using peer or non-peer faces should be a theoretical consideration of future research because the same emotion displayed on non-peers’ compared to peers’ faces may have different implications and meanings to the perceiver.

## Introduction

A growing number of studies (Anastasi and Rhodes, 2006; Kuefner et al., 2008; Hills and Lewis, 2011; Rhodes and Anastasi, 2012; Macchi Cassia et al., 2015) point out that both children and adults exhibit poorer performance recognizing non-peer compared to peer faces. If there is such a bias, it seems plausible that it also affects the recognition of emotional cues on the faces. Previous studies (Calvo and Nummenmaa, 2008; Purcell and Stewart, 2010; Becker et al., 2011; Tamm et al., 2017; Becker and Rheem, 2020) listed several possible confounding variables that might be responsible for mixed results in visual search tasks for emotional facial expressions both in children and adults. Most studies point out several possible sources of confound in the facial stimuli, leading to incorrect inferences; such confounding factors were found in both photographs and schematic faces. For instance, Purcell et al. (1996) first noted that photographs’ illumination, brightness, and contrast artifacts were to be carefully controlled. The use of schematic faces cannot be the answer (Coelho et al., 2011; Beaudry et al., 2014; Watier, 2018, see also Kennett and Wallis, 2019 for more recent findings) as—beyond violating ecological validity—they also comprise low-level visual features related to the interaction between the lines representing eyebrows and mouth and the surroundings representing the head, forming “T” junctions with the surround. These junctions and differences in line orientation are likely responsible for the search advantage seen in schematic stimuli rather than the “emotions” displayed on the faces. Nonetheless, previous research aiming to discover which emotional facial expression (i.e., anger or happiness) has an advantage in visual processing in preschool children mostly used adult faces (Silvia et al., 2006; Waters and Lipp, 2008; Waters et al., 2008; Lobue, 2009; Farran et al., 2011; Rosset et al., 2011). Thus, the goal of this study is to address another potential confounding variable, the own-age bias (OAB), which was rarely, if at all, acknowledged in previous experiments. The OAB refers to a phenomenon in which individuals exhibit reduced performance in recognizing and detecting expressions of emotions on faces that are either much younger or much older than themselves (Anastasi and Rhodes, 2005, 2006; Kuefner et al., 2008; Hills and Lewis, 2011; Riediger et al., 2011; Rhodes and Anastasi, 2012; Marusak et al., 2013; Craig et al., 2014b; Macchi Cassia et al., 2015).

From an evolutionary point of view, prioritized detection for emotional cues on the faces of others was likely adaptive throughout human evolution as it helped survival by, for instance, foreshadowing danger (angry expression), or promoting trustworthiness (happy expression). Several research fronts are focused on the root causes of visual search advantage related to schematic faces (Kennett and Wallis, 2019), emotional interpretation of Emojis (Franco and Fugate, 2020), or comparing different methods related to happy faces search advantage research (Rohr et al., 2012; Calvo and Beltrán, 2013; Wirth and Wentura, 2020). However, whether happy or angry emotional facial expressions portray particular features that foster accurate and prompt recognition and detection is still debated (e.g., Calvo and Nummenmaa, 2008; Becker and Rheem, 2020). One of the first theories, the anger superiority effect (ASE), demonstrated a fast detection of angry faces when presented in a visual search task—the face in the crowd paradigm (Hansen and Hansen, 1988; Lundqvist et al., 1999; Öhman et al., 2001). It has been argued that this search advantage was a key factor in survival as the advantaged recognition of anger on faces provided a warning that aversive consequences were likely to follow, and thus, gave the perceiver’s CNS more time to prepare a fight or flight response (Öhman et al., 2001). These studies (e.g., Hansen and Hansen, 1988; Öhman et al., 2001) often used a 3 × 3 matrix array with nine (adult) faces each trial, one of them displaying a different emotion than the other eight. Participants’ (adults) task was to detect if discrepant faces were present and respond (yes/no) as quickly as possible on a keyboard or by pointing the target’s position through the use of a touchscreen. Many of these studies concluded that angry faces pop out of the crowd; i.e., they were found much faster than happy, sad, or fearful facial expressions. More recently, the ASE has been shown with a large sample of preschool children using color photographs and schematic adult faces (Lobue, 2009; LoBue et al., 2014). Results in various clinical populations lend further support for the ASE, e.g., children suffering from different developmental issues, such as autism spectrum disorder and Williams Syndrome (Rosset et al., 2011) as well as adults suffering from Asperger Syndrome (Ashwin et al., 2006). However, the results are not consistent.

In parallel to the ASE, numerous other studies (Kirita and Endo, 1995; Juth et al., 2005; Miyazawa and Iwasaki, 2010; Svärd et al., 2012; Craig et al., 2014a; Nummenmaa and Calvo, 2015; Lee and Kim, 2017, see also Pool et al., 2016 for review) found a search advantage to happy faces compared to negative emotional expressions in adults using faces of adults. This was named the happiness superiority effect (HSE). The HSE asserts that the quick detection of happy faces conveys an adaptive function to maximize social reward and foster alliances and collaborations (Calvo and Nummenmaa, 2008). Furthermore, it has been shown that processing happy faces requires less attentional resources compared to anger and other expressions (Becker et al., 2011; Pool et al., 2016). Studies demonstrating the HSE use a similar visual search task to the ASE. Furthermore, HSE has also been shown in children with different developmental or personality disorders, such as autism spectrum disorder (Farran et al., 2011) and social anxiety (Silvia et al., 2006).

Most of the aforementioned research in preschool children examining the advantage of emotional facial expressions in visual search tasks used mainly adult faces as targets (Silvia et al., 2006; Waters and Lipp, 2008; Waters et al., 2008; Lobue, 2009; Farran et al., 2011; Rosset et al., 2011). The perceived intentions of a peer relative to either another peer (e.g., another child) or non-peer (e.g., an adult) showing a similar expression can very well differ (Rhodes and Anastasi, 2012; Marusak et al., 2016). Therefore, the interpretation of the expression might be different when displayed on the faces of people of different ages (Craig et al., 2014b). Indeed, due to the emergence of social competence needs in preschool children, emotional cues from peers become more critical than that of non-peers (Trentacosta and Fine, 2010). To the best of our knowledge, this is the first study systematically examining the recognition of emotional facial expressions in a sample of children using faces of age-matched peers and non-peers.

Therefore, our overarching goal of the present study was to test ASE vs. HSE on two independent samples of preschool children and adults, using photos of children as well as adult faces displaying emotions. Considering this age gap variable, the matrix of interactions comprises (a) a child observing another child’s facial expression, (b) a child observing an adult, (c) an adult observing another adult, and (d) an adult observing a child. When (a) a child observes another child’s angry face, anger might imply social rejection and even some danger as children might hurt each other. In contrast, when (b) an adult angry face is shown to a child, it may signal authority or a reprimand, for example, related to something necessary. However, it can also signal violence (White et al., 2019). In fact, children have been shown to have stronger amygdala activation in response to angry adult faces compared to angry child faces (Hoehl et al., 2010). Albeit the survival value of quickly detecting angry facial expressions, regarding children, we expected a HSE, as peer connections have growing importance at this age (e.g., Craig et al., 2014a). Furthermore, recognition expertise and speed depend on the extent of exposure to certain groups; e.g., children may see other children particularly often in kindergartens. In contrast, they less frequently see a lot of older people (Anastasi and Rhodes, 2005). Moreover, happy faces are seen more often in general, which might lead to an added expertise effect (Calvo et al., 2014). In (c), an adult–adult situation, the angry face can convey a more grave reason, with the potential for severe consequences (Öhman and Dimberg, 1978; Hansen and Hansen, 1988). Among adults, happy expressions can signal a variety of intentions that are relevant to the observer, such as acceptance, affiliation, collaboration, safety, trustworthiness, and even sexual attraction. In contrast, when (d) an adult sees an angry expression on a child’s face, they might not find anger on a child’s face threatening (Hoehl et al., 2010). However, fearful signals on a child’s face could mean a need for attention and calls for comfort and care. Regarding happy expressions, a child’s happiness is a strong positive feedback and would signal contentment toward the adult (Ahn and Stifter, 2006).

Due to the versatility of the happy facial expression and its possible interpretations, happy faces are expected to present the highest search advantage in both adults and children, regardless of the age of the faces. Moreover, happy faces are also more frequently observed, leading to familiarity (Calvo et al., 2014). In addition, a recent meta-analysis (Pool et al., 2016) have shown that positive stimuli are particularly able to recruit more attentional resources when the individual is motivated to obtain the positive stimulus, and despite the presence of concurrent competing stimuli (see also Gable and Harmon-Jones, 2008; Harmon-Jones and Gable, 2009). If attention is automatically oriented toward stimuli that are motivationally relevant for the temporary goal of the individual (Vogt et al., 2013; Mazzietti et al., 2014), it might be more likely that people attend faster to happy faces as these not only are usually non-threatening but also often bring some reward and represent a friendly and accepting environment.

## Materials and Methods

The paradigm used in this paper is similar to previous studies developed to test the attentional bias toward emotional facial expression (e.g., Hansen and Hansen, 1988; Eastwood et al., 2001; Calvo and Nummenmaa, 2008). Participants were asked to observe nine pictures at a time in a 3 × 3 block arrangement. The facial expression in one of the pictures, *the target*, was different from the others, i.e., *the crowd*. All pictures presented in one trial were of different people. In line with previous studies (Juth et al., 2005), we used angry, happy, and fearful faces as targets and neutral faces as a crowd.

However, our paradigm includes a critical novelty. Both children and adults completed the experiments. We used color photographs of children and adults of similar age to our participant groups, instead of faces of adults only or schematic pictures. A 3 × 2 × 2 design was used with emotion (i.e., angry, happy, and fearful) as a within-subject factor and Group (adult or child) and Model (adult or child) as between-subject factors. The gender of models was balanced to present a female target in a female crowd in half of the trials, and a male target in a male crowd, in the other half.

The control of low-level features is particularly necessary when studying a single group of participants responding to several types of emotion. If not well-controlled, one might erroneously find a misleading result, suggesting, for example, that particular emotion is more rapidly processed, when in fact, the angry pictures were a bit brighter by chance due to the eyes being more open or the teeth more showing. This study used a large number of pictures (70 adults and 80 children) controlled for color brightness, contrast, spatial frequency, and luminance values; exposed teeth ratio; and used two participant groups to minimize error.

Our research was approved by the local Ethical Review Committee for Research in Psychology (Nr. 2018-62) and carried out following the Code of Ethics of the World Medical Association (Declaration of Helsinki). Informed written consent was obtained from adult participants and parents of children participants, and oral consent was obtained from the children.

### Participants

The exclusion criteria for the current study were a history of depression, anxiety, or a neurodevelopmental disorder^1^. The inclusion criterion was the successful completion of an emotion labeling task. First, participants were shown the faces used in the experiment and asked to name the emotions displayed on them. If they reached a success rate of 70%, *a priori* set by the authors, separately for each emotion, could progress to the visual search task^2^. If not, they were told that the experiment is over; children could choose a small gift for their participation. Including only those who could correctly identify the facial expressions was essential to reduce confounding biases and variance due to false recognition.

A total of 146 volunteers participated in our study (78 children and 68 adults); about half of them completed the visual search task with faces of children, while the other half completed the visual search task with faces of adults. This design resulted in a total of four groups: (1) children who completed the visual search task seeing faces of children (*n* = 43) or (2) adults (*n* = 34), and (3) adults who completed the task with faces of children (*n* = 37) or (4) adults (*n* = 31). See details about the groups in the next two sections *“Visual search with faces of children”* and “*Visual search with faces of adults*”). The sample size for our study was determined by computing the estimated statistical power based on the effect sizes of prior experiments on HSE and ASE using a similar task (Hansen and Hansen, 1988; Eastwood et al., 2001; Juth et al., 2005; Lobue, 2009; LoBue et al., 2014). For this estimation, we used the G^∗^power 3 software (Faul et al., 2007). The initial analysis was based on previous results (β = 0.8, *f* = 0.40, and a correlation between measures = 0.5), indicating that a total sample size of 12 would provide sufficient statistical power. A more conservative estimation (β = 0.95, *f* = 0.25, and *r* = 0.35) indicated that a total sample size of 76 is required. Therefore, we collected nearly double the required sample size. The *post hoc* analysis showed that the achieved power in this study was 0.99.

#### Visual Search With Faces of Children

Initially, 56 children were recruited. However, 12 children failed to reach *a priori* criteria (70% success rate) and, therefore, were excluded. Forty-three (19 boys, 24 girls) preschool children completed the visual search task. Their mean age was 5.65 years (SD = 0.78). Their ability to name the facial expressions displayed on the photographs used was tested (see section *“Procedure”*). The mean success rate for this sample was 91.71% (SD = 21.50%). The mean success rate for the whole sample was 72.88% (SD = 33.63%).

The adult sample comprised 37 (13 males and 24 females) adults with a mean age of 21.8 years (SD = 0.78). All of the adults passed the *a priori* criteria of naming the facial expressions (*M* = 96.33%, SD = 12.27%). All of the participants were Caucasian, right-handed, with normal to corrected-to-normal vision.

#### Visual Search With Faces of Adults

Initially, we recruited 44 children. However, 10 children failed to reach *a priori* criteria (70% success rate) and were, therefore, excluded. The children tested sample comprised 34 (14 boys and 20 girls) preschool children who completed the visual search task. Their mean age was 5.47 years (SD = 0.75). The mean success rate of naming the facial expressions for this sample was 90.57% (SD = 20.90%). The mean success rate for the whole sample was 71.23% (SD = 34.68%).

The adult sample comprised 31 (12 males and 19 females) adults with a mean age of 21.1 years (SD = 1.31). All of the adults passed the *a priori* criteria of naming the facial expressions (*M* = 98.67%, SD = 5.46%). All of the participants were Caucasian right-handed with normal or corrected-to-normal vision.

### Stimuli

#### Child’s Faces

All of the pictures (target and crowd) were taken from the Dartmouth Database of Children’s Faces (Dalrymple et al., 2013). The Dartmouth Database contains a set of photographs of 80 Caucasian children between 6 and 16 years of age, each of whom displaying eight different facial emotions. Photographs were taken from the same angle, frontal view, and under the same lighting condition. The models in the database are wearing black hats and black gowns to minimize extra-facial variables. We selected 18 child models (9 girls), ranging in ages between 6 and 10 years (estimated age: 5.7–8.6), and three emotions as target expressions (angry, happy, and fearful). Photographs with neutral expressions served as a crowd. Past studies (Calvo and Nummenmaa, 2008) have warned that exposed teeth could produce high local luminance, increasing physical saliency, and, thus, attract attention and facilitate detection based on physical and not emotional saliency. In our study, there was no difference between the ratio of expressions with exposed and concealed teeth among the three emotion categories (χ^2^ < 1, *p* > 0.1).

#### Adult Faces

All of the pictures (target and crowd) were taken from the Karolinska Directed Emotional Faces database (Lundqvist et al., 1998). The database contains a set of photographs of 70 Caucasian individuals, each displaying seven different emotional expressions, each expression being photographed twice from five different angles and under the same lighting condition. We only used frontal views similarly to Exp1. We selected photographs with three emotions as target expressions (angry, happy, and fearful). The pictures exposed teeth ratio was matched as used in Exp1 until no difference was observed between the ratio of expressions with exposed and concealed teeth among the three emotion categories (χ^2^ < 1, *p* > 0.1). Again, photographs with neutral expressions served as the crowd.

### Visual Display and Apparatus

The 3 × 3 sets were created in a block arrangement (measuring 22.45° × 22.45° in total), with eight crowd members (measuring 7.57° × 7.57° each), and one target (same size as background pictures). Images were separated with a 2pt wide black border. All three target emotions were presented in each of the nine possible locations, separately for boy and girl models. Thus, the stimuli set consisted of 54 matrices, i.e., nine trials of each of the six conditions. The male and female trials were presented separately, in 27-trial blocks, counterbalanced over participants, i.e., half of the respondents started with male trials, the other half started with female trials.

Although visual search paradigms can be sensitive to potential low-level confounds, face color and original appearance were retained for ecological validity. We calculated color brightness, contrast, spatial frequency, and luminance values for each matrix using Matlab after data collection to monitor whether these values could be a source of bias. We found no significant difference between the calculated low-level visual feature values within the pictures. Furthermore, these values had no covariate effects on the results (all *F*s < 1, *p* > 0.1).

The stimuli appeared on a 17-inch LCD touchscreen color monitor with a visible area of 15 inches and a resolution of 1,366 × 768, refresh rate, and a sampling rate of 60 Hz, 24-bit color format. The stimuli set were presented using PsychoPy Software version 1.83 for Windows (Peirce, 2007).

### Procedure

All participants (children and adults) completed the same procedure. Those who passed the emotion labeling task were taught how to use the touchscreen monitor if it was necessary. In the case of children, the experimenter helped them to create a drawing of their right hand on a sheet of paper. For adults, we used a previously printed and laminated paper that had the outline of a hand on it. Participants were asked to place their right hand on this paper between trials. Children were seated approximately 30 cm and adults approximately 60 cm in front of the monitor. First, all participants completed nine practice trial matrices, one with each target. Responses to practice trials were excluded from further analyses. If the experimenter saw that they understood the task, and the participants also gave their oral consent to continue, the experiment started. Respondents completed the task in two sessions with a short break between them. The instruction was to find the picture that shows a different emotional expression from the others as quickly as possible. For children, the experimenter started each stimulus by hitting a button on the keyboard; for adults, the images automatically appeared (with 1 s interstimulus interval). Each image was preceded by a cartoon figure (for children) or a fixation cross (for adults) presented for 500 ms. Then, the participant indicated the target’s location by touching it on the touchscreen monitor. Upon completing the experiment, children could choose a small gift as a reward for their efforts. The task lasted for approximately 15–20 min, including the break between the two blocks^3^.

### Data Analyses

Trials with very low pointing accuracy (i.e., above the two-standard-deviation criterion on the raw data of coordinates) or very high reaction time values (two standard deviations from the mean) were excluded from further analyses, comprising less than 1% of the data. The statistical analyses were performed using the JAMOVI Statistics Program Version 0.9. for Windows (Jamovi Project, 2018). Reaction times were averaged over trials yielding three variables—Emotion (i.e., angry, happy, and fearful)—for all groups: Group (children vs. adults) × Model (children vs. adults). These variables were then entered into a 3 × 2 × 2 mixed ANOVA with emotion as a fixed factor; Group and Model as independent factors.

## Results

### The Effect of Emotion on Search Performance

The main effect of emotion was found significant [*F*(2,284) = 64.89, *p* < 0.001, and η_*p*_^2^ = 0.31]. The Tukey corrected pairwise comparisons revealed that participants found happy faces (*M* = 3.14 s, 95% CI = 3.00–3.27) faster than angry [*M* = 3.62 s, 95% CI = 3.49–3.75; *t*(284) = 8.66, and *p* < 0.001] and fearful faces [*M* = 3.74 s, 95% CI = 3.60–3.87; *t*(284) = 10.74, and *p* < 0.001], but the latter two did not differ [*t*(284) = 2.08, *p* = 0.095].

**FIGURE 1:**
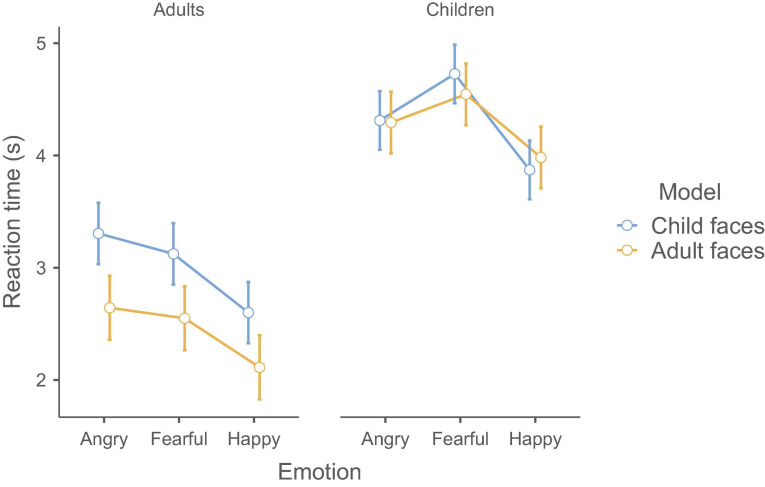
The three-way interaction between Emotion, Group, and Model. The significant effects were the main effect of Emotion, Group, and Model as well as the two-way interactions between Emotion and Group and between Group and Model. The results are in seconds; 95% confidence intervals are shown.

We also found a two-way interaction between Emotion and Group [*F*(2,284) = 9.08, *p* < 0.001, and η_*p*_^2^ = 0.06]. To tease apart this interaction, we used two separate ANOVAs for children and adults. These follow-up analyses revealed that, for adults, the main effect of emotion was significant [*F*(2,140) = 61.23, *p* < 0.001, and η_*p*_^2^ = 0.47] with the same pattern as reported above; i.e., participants found happy faces faster than angry [*t*(140) = 10.47, *p* < 0.001] and fearful faces [*t*(140) = 8.34, *p* < 0.001], while the latter two did not differ [*t*(140) = 2.13, *p* = 0.087]. For children, the main effect was also significant [*F*(2,144) = 29.09, *p* < 0.001, and η_*p*_^2^ = 0.29]. However, the pattern was different: Children found happy faces faster than angry [*t*(144) = 3.90, *p* < 0.0001] and fearful faces [*t*(144) = 7.63, *p* < 0.001] and also angry faces were found faster than fearful ones [*t*(144) = 3.73, *p* < 0.001]. See Figure 1 for the interaction and Table 1 for detailed descriptive statistics.

**TABLE 1:** Descriptive statistics of the search times in seconds for child and adult participants’ search task (angry, fearful, and happy) on faces of children and adults.

				95% Confidence interval	
Group	Model	Emotion	Mean	Lower	Upper
Adults**	Child’s faces*	Angry	3.28	3.02	3.54
		Fearful	3.14	2.88	3.39
		Happy**	2.60	2.34	2.85
	Adult faces*	Angry	2.62	2.34	2.90
		Fearful	2.52	2.25	2.80
		Happy**	2.11	1.83	2.39
Children**	Child’s faces	Angry**	4.26	4.00	4.51
		Fearful**	4.71	4.45	4.97
		Happy**	3.85	3.59	4.10
	Adult faces	Angry**	4.32	4.05	4.59
		Fearful**	4.57	4.30	4.84
		Happy**	3.99	3.72	4.26

Mean scores and confidence intervals are in seconds.*p < 0.05, **p < 0.001; and the value differs from the other categories.

The other interactions involving Emotion were non-significant, i.e., between Emotion and Model [*F*(2,284) = 1.64, *p* = 0.196] and between Emotion and Model and Group [*F*(2,284) = 0.62, *p* = 0.539].

### The Effects of Model and Group

The main effect of the Group was significant [*F*(1,142) = 174.55, *p* < 0.001, and η_*p*_^2^ = 0.55], meaning that adults (*M* = 2.71 s, 95% CI = 2.55–2.88) were generally faster than children (*M* = 4.28 s, 95% CI = 4.12–4.45) in both tasks. Furthermore, the main effect of the Model was also significant [*F*(1,142) = 5.61, *p* = 0.019, and η_*p*_^2^ = 0.04] showing that participants were faster to identify the emotions on faces of adults (*M* = 3.36 s, 95% CI = 3.19–3.52) compared to faces of children (*M* = 3.64 s, 95% CI = 3.47–3.80).

The two-way interaction between Group and Model was significant [*F*(1,142) = 6.56, *p* = 0.011, and η_*p*_^2^ = 0.04], revealing that the main effect of the Model was only significant for adults but not for children. Interestingly, children detected emotions on the faces of adults (*M* = 4.29 s, 95% CI = 4.06–4.53) and faces of children (*M* = 4.27 s, 95% CI = 4.04–4.50) at similar speeds. In contrast, adults were faster to detect emotions on the faces of adults (*M* = 2.42 s, 95% CI = 2.18–2.66) compared to faces of children (*M* = 3.00 s, 95% CI = 2.78–3.23).

## Discussion

The present study sought to test visual search advantages for angry and happy faces, i.e., to compare ASE with HSE theories, respectively. Past research has shown mixed results, and an important possible confounding variable—namely the OAB—has been overlooked in previous research designs, as children usually completed the visual search for emotional expressions on the faces of adults. However, the emotions could portray very different meanings based on who sees it (a child or an adult) on whose face (a peer or a nonpeer). For example, previous research (Thomas et al., 2001; Adolphs, 2010; Marusak et al., 2013) has shown different activation patterns in the amygdala in children and adults while viewing adult faces. Therefore, we recruited a pool of children and adults who performed a classical visual search task with children and adult models showing various facial expressions. We found that children and adults found happy faces significantly faster than angry and fearful faces regardless of it being present on the faces of peers or non-peers. This is compatible with the notion of previous results showing an attentional bias for positive emotional stimuli (Brosch et al., 2011; Pool et al., 2016; Wirth and Wentura, 2020). Furthermore, we did not find clear evidence for an OAB regarding the visual search of emotional expressions; there were only some differences between adults and children searching among faces of peers and non-peers. The reason behind this might be that it has been shown that very similar neural networks were implicated in the processing of angry and happy faces, in both adults and children (Hoehl et al., 2010). Including children and adults in the study, performing as both model and participant, allowed us to explore OAB in a face in the crowd “scenario.” This was a particular strength of our study, and data showed significant differences between the peer and non-peer faces.

Overall, our findings support the HSE across both samples. That is, faces displaying a happy expression were found quicker in a visual search task and had an advantage in visual processing corroborating previous studies (Hoehl et al., 2010) using adult faces in testing adult participants (Kirita and Endo, 1995; Nummenmaa and Calvo, 2015; Savage et al., 2016) and children (Silvia et al., 2006; Farran et al., 2011; Lagattuta and Kramer, 2017). In peer relations (for both children and adults), the happy expression could be seen as a powerful social tool to communicate friendly intent and show assurance and acceptance (Juth et al., 2005). Moreover, a child’s happiness would signal contentment toward the adult and strong positive feedback (Ahn and Stifter, 2006). Also, an adult showing happiness toward children could mean, for instance, reward, reinforcement, friendly intent, and safety (McClure, 2000). Furthermore, trustworthiness is also highly associated with happy expression (Calvo et al., 2017). Hence, the processing advantage of happy faces is adaptive due to its importance in social situations, such as reconciliation, sharing, and collaborations (Calvo and Nummenmaa, 2008; Becker et al., 2011). Indeed, it has been shown (LaBarbera et al., 1976; Walden and Field, 1982; Striano et al., 2002; Grossmann et al., 2007) that children identify happy expressions earlier in development and more reliably. Regarding the cognitive mechanisms underlying these results, it has been argued that (1) the procession of angry faces requires less attentional resources compared to angry and other expressions (Becker et al., 2011; Pool et al., 2016) and, furthermore, that (2) happy faces facilitate global processing while angry expressions facilitate local processing (Kerusauskaite et al., 2020). However, the HSE may also reflect a positivity bias due to our expectations of positive over negative signals (Leppänen and Hietanen, 2004), i.e., facilitating the recognition of happy faces. Another possible explanation may be driven by the relative occurring frequency of each emotional expression in social encounters (Calvo et al., 2014). Calvo and colleagues have shown that happy faces are seen more often, leading to more exposure that adds expertise to the detection of these expressions. They found that happy faces are seen more often and detected faster than angry faces and that angry faces are seen more often and detected faster than fearful faces, which also corroborates our findings. Similarly, OAB might be explained by natural visual statistics alone, because adults and children may differ in exposure frequency to faces of adults or children and also to the different emotions (for a more detailed review, see Calvo and Nummenmaa, 2008).

Adults also found happy faces first; nonetheless, we only found a marginally significant effect showing they reacted to angry faces slower than fearful faces regardless of the age of the model. It is likely that adults did not find anger on a child’s face as threatening (Hoehl et al., 2010). Presumably, adults are more attuned to recognize and attend to children’s fearful expressions. Fearful signals on a child’s face could mean a need for attention, protection, and calls for comfort as the child might be in pain or need of care. Fear can also alert the perceiver to danger in the environment (Thomas et al., 2001) and can also be perceived as affiliative and appeasing and preventing aggressive encounters and reduce the likelihood of injuries (Marsh et al., 2005). As for adults detecting fear on other adult’s faces, our results are somewhat contradictory to previous findings. Yet, a fearful face can signal an indirect threat that the viewer is not aware of, which in turn could facilitate visual search performance and preattentive capture of attention (Bannerman et al., 2009; Pritsch et al., 2017).

In children, anger was detected faster than the fearful expression in line with our expectations. When anger is present on an adult’s face, it could signal reprimand or even aggression, triggering a quick defensive reaction (LeDoux and Daw, 2018; White et al., 2019). When it is present on another peer’s face, anger can signal rejection. Peer connections become more important around this age (Trentacosta and Fine, 2010), and emotional cues of acceptance and rejection from peers turn out more critical compared to other expressions. Hence, the detection of fearful faces was the slowest, which is similar to previous research in adults using eye-tracking measures (Wells et al., 2016).

We also found that adults detected emotions in other adults’ compared to children’s faces faster, an effect we did not observe in children. On the one hand, this could lend further support to the notion that the OAB is likely not the product of familiarity as older adults have necessarily previously been members of other age groups (Anastasi and Rhodes, 2005). Adult faces are associated with enhanced neural processing (Marusak et al., 2013) and children appear to be more accurate at recognizing faces of adults (Macchi Cassia, 2011). However, people such as school teachers interact with children more frequently and show improved capacity to recognize child faces (Harrison and Hole, 2009). Our adult sample consisted of participants who were rather young and did not have children and they supposedly did not have daily contact with preschool children, while children did have daily contact with adults. We think that the frequency effect (Calvo et al., 2014) could explain this finding as well.

Some limitations of this study shall be noted. First, we used only three emotional expressions based on previous studies investigating CFE and HSE. Nonetheless, including all basic emotions would be necessary for future studies, particularly neutral faces as controls. The visual search paradigm adopted in our study made it impossible to include a neutral condition. Prioritization of a specific emotion may be task-dependent (see also Cisler et al., 2009; Zsido et al., 2019). Also, there is some evidence showing that, under some circumstances, neutral facial expressions may be evaluated as negative (Lee et al., 2008). The fact that, in this study, all participants successfully categorized neutral faces as neutral in the pre-test might point to the notion that this issue was not present. Changing the expression of the faces in the crowd in future experiments might also carry interesting theoretical implications. Although steps were taken to control for visual confounds by the creators of Dartmouth and the Karolinska Directed Emotional Faces databases, the photographs used were not originally averaged on low-level visual features. Although our study is a promising first step in controlling for an often-neglected factor, future studies are needed to explore the effects of OAB on the detection of emotional expressions. Finally, our results could be explained by simple natural visual statistics; i.e., adults and children may differ in exposure frequency to faces of adults or children and also to the different emotions. Nevertheless, OAB still stands as a bias to be controlled in future studies.

Despite these limitations, to our knowledge, this is the first study to demonstrate the happiness advantage effect in both children and adults using peer’s and non-peer’s faces. That is, young children found happy faces quicker compared to angry and fearful ones. This novel developmental evidence might add further support to the robustness and reliability of the HSE. Overall, this finding contributes to the understanding of the differences in detecting emotional faces. Using peer or non-peer faces should be a theoretical consideration of future studies because results will change based on the choice as emotions seen on either peer or non-peer faces have different implications and meanings to the perceiver.

## Data Availability Statement

The raw data supporting the conclusions of this article will be made available by the authors, without undue reservation.

## Ethics Statement

The studies involving human participants were reviewed and approved by Hungarian Ethical Review Committee for Research in Psychology. Written informed consent to participate in this study was provided by the participants’ legal guardian/next of kin.

## Author Contributions

AZ, VI, JB, and AS: conceptualization. AZ, NA, OI, BL, and CC: methodology. AZ, NA, OI, VI, and JB: formal analysis and investigation. AZ, NA, VI, JB, TM-B, OI, AS, BL, and CC: writing—original draft preparation. AZ, NA, VI, JB, TM-B, OI, AS, BL, and CC: writing—review and editing. AZ, NA, TM-B, OI, BL, and CC: funding acquisition. NA, VI, JB, TM-B, and OI: resources. AZ, AS, BL, and CC: supervision. All authors contributed to the article and approved the submitted version.

## Conflict of Interest

The authors declare that the research was conducted in the absence of any commercial or financial relationships that could be construed as a potential conflict of interest.

## Footnotes

1 Adults filled out the trait subscale of the short, five-item version of the Spielberger State-Trait Anxiety Inventory (Zsido et al., 2020) and were asked to report if they had a history of depression, while in the case of children, we asked their parents to do so. Participants did not know we are asking these questions as an inclusion criterion.

2 We also used Pearson correlation to test the correlation between reaction time and emotion knowledge indicated by the success rate on the emotion naming task in children. When children saw faces of other children, we found a significant correlation between reaction time and emotional knowledge (*r* = −0.33, *p* = 0.032), indicating that children who performed better on the emotion naming task were generally faster to find the emotional faces. The correlation between reaction time and emotional knowledge (*r* = -0.29, *p* = 0.096) was non-significant, indicating a similar trend when children searched among faces of adults.

3 Children’s performance can be sensitive to load; i.e., it may change during the experiment. Although we did not intend to measure the effects of cognitive load and we used two blocks with a short break in-between to avoid any effect of overwhelming the children, one of the reviewers pointed out that it might still be feasible to check the time course data. Therefore, we used Spearman correlations to test whether reaction time changed over time. We found no indication of effect of load (rho values < |0.1|). Further, the visual check confirmed that there was no non-linear connection either.
